# JYNNEOS Vaccination Coverage Among Persons at Risk for Mpox — United States, May 22, 2022–January 31, 2023

**DOI:** 10.15585/mmwr.mm7213a4

**Published:** 2023-03-31

**Authors:** Lauren E. Owens, Dustin W. Currie, Ellen A. Kramarow, Safana Siddique, Megan Swanson, Rosalind J. Carter, Jennifer L. Kriss, Peter M. Boersma, Florence C. Lee, Ian Spicknall, Elizabeth Hurley, Maria Zlotorzynska, Adi V. Gundlapalli

**Affiliations:** 1 CDC Mpox Emergency Response Team.

From May 2022 through the end of January 2023, approximately 30,000 cases of monkeypox (mpox) have been reported in the United States and >86,000 cases reported internationally.* JYNNEOS (Modified Vaccinia Ankara vaccine, Bavarian Nordic) is recommended for subcutaneous administration to persons at increased risk for mpox (*1*,*2*) and has been demonstrated to provide protection against infection (*3*–*5*). To increase the total number of vaccine doses available, the Food and Drug Administration (FDA) issued an Emergency Use Authorization (EUA) on August 9, 2022, recommending administration of the vaccine intradermally (0.1 mL per dose) for persons aged ≥18 years who are recommended to receive it (*6*); intradermal administration can generate an equivalent immune response to that achieved through subcutaneous injection using approximately one fifth the subcutaneous dose (*7*). CDC analyzed JYNNEOS vaccine administration data submitted to CDC from jurisdictional immunization information systems (IIS)^†^ to assess the impact of the EUA and to estimate vaccination coverage among the population at risk for mpox. During May 22, 2022–January 31, 2023, a total of 1,189,651 JYNNEOS doses (734,510 first doses and 452,884 second doses)^§^ were administered. Through the week of August 20, 2022, the predominant route of administration was subcutaneous, after which intradermal administration became predominant, in accordance with FDA guidance. As of January 31, 2023, 1-dose and 2-dose (full vaccination) coverage among persons at risk for mpox is estimated to have reached 36.7% and 22.7%, respectively. Despite a steady decline in mpox cases from a 7-day daily average of more than 400 cases on August 1, 2022, to five cases on January 31, 2023, vaccination for persons at risk for mpox continues to be recommended (*1*). Targeted outreach and continued access to and availability of mpox vaccines to persons at risk are important to help prevent and minimize the impact of a resurgence of mpox.

Since the beginning of the 2022 mpox outbreak through January 31, 2023, a total of 30,157 mpox cases and 32 associated deaths have been reported in the United States.^¶^ Most *Monkeypox virus* infections during the current outbreak have been transmitted through close, intimate contact (primarily sexual) with symptomatic persons (*8*). Based on the epidemiology of the current outbreak, CDC recommends mpox vaccination for persons at increased risk for mpox, including, but not limited to 1) persons with known or presumed exposure; 2) gay, bisexual, or other men who have sex with men (MSM) and transgender, nonbinary, or gender-diverse persons with multiple recent sexual partners; 3) MSM and transgender, nonbinary, or gender-diverse persons with a newly diagnosed sexually transmitted disease; 4) persons who have had sex at or related to attending a commercial sex venue or another large social-cultural gathering during the previous 6 months; 5) those with sexual partners with any of the aforementioned risks; and 6) persons with HIV or other causes of immune suppression who have had recent or anticipate future *Monkeypox virus* exposure through any of these scenarios.** Preliminary studies have indicated that JYNNEOS provides protection against mpox, with unvaccinated persons having 7–14 times higher incidence than do vaccinated persons, depending on the number of doses received (*3*–*5*).

Health care providers submitted mpox vaccination data to their jurisdictions’ IIS; these data were then submitted to CDC. IIS data include information about the vaccine (e.g., manufacturer, dose, and administration route), recipient (e.g., age, sex,^††^ race and ethnicity, and residence), and provider (location and provider type). This analysis includes data from JYNNEOS vaccine administered to residents in the 50 U.S. states, the District of Columbia (DC), New York City, Philadelphia, Puerto Rico, the U.S. Virgin Islands, Guam, and the Northern Mariana Islands, during May 22, 2022–January 31, 2023.

JYNNEOS vaccination coverage (the estimated proportion of the population recommended for vaccination that has been vaccinated) was calculated by jurisdiction.^§§^ The numerator used in calculating each jurisdiction’s coverage included data submitted to CDC on all persons aged ≥13 years with valid residence information in each jurisdiction who have been partially or fully vaccinated^¶¶^ with JYNNEOS. The denominator, representing the population at increased risk for *Monkeypox virus* exposure (*8*), was estimated as the number of MSM who were indicated to receive HIV preexposure prophylaxis (PrEP) (among those aged ≥16 years) plus the number of MSM with HIV (among those aged ≥13 years) in each jurisdiction, using data that are publicly available via CDC AtlasPlus.*** The estimated population was then increased by 25% for each jurisdiction to account for additional persons eligible for vaccination (e.g., MSM who are at increased risk for mpox but do not have indications for PrEP, cis-female or transgender partners of MSM, close contacts [of any age] of persons with known or suspected mpox, and persons at risk for occupational exposure to orthopoxviruses)^†††^ (Supplementary Table; https://stacks.cdc.gov/view/cdc/126171).^§§§^ This activity was reviewed by CDC and was conducted consistent with applicable federal law and CDC policy.^¶¶¶^

During May 22, 2022–January 31, 2023, 57 jurisdictions administered a total of 1,189,651 doses of JYNNEOS vaccine, 734,510 (61.7%) of which were first doses, 452,884 (38.1%) of which were second doses, and 2,257 (0.2%) of which were reported as dose 3 or higher (Table). The majority of the 734,510 persons who received ≥1 dose of vaccine were male (91.4%) and aged 25–49 years (64.2%). Among the 91% of first-dose recipients for whom race and ethnicity data were available, 51.2% were non-Hispanic White, 22.7% were Hispanic or Latino, 12.4% were non-Hispanic Black or African American (Black), 7.4% were non-Hispanic Asian, 5.6% were non-Hispanic multiracial, and <1% were non-Hispanic American Indian or Alaska Native and non-Hispanic Native Hawaiian or other Pacific Islander. The most common vaccination locations included public health clinics (39.5%), commercial vaccination service providers (13.4%),**** medical practices (10.2%), hospitals (8.8%), and Federally Qualified Health Centers (6.2%). The highest number of both first and second doses were administered to persons living in the West U.S. Census Bureau region (261,936 and 160,457 doses, respectively); >80% of all vaccine recipients reported living in counties categorized as urban.

**TABLE:** Characteristics of persons who received first and second doses of JYNNEOS vaccine — United States, May 22, 2022–January 31, 2023*

Characteristic	No. (%)^†^	
	First dose^§^	Second dose
**Total**	**734,510 (100)**	**452,884 (100)**
**Sex**		
Female	62,191 (8.6)	28,066 (6.3)
Male	659,659 (91.4)	419,140 (93.7)
Unknown	12,660 (—)	5,638 (—)
**Age group, yrs**		
0–4	302 (0.04)	66 (0.01)
5–11	406 (0.06)	130 (0.03)
12–17	586 (0.08)	216 (0.05)
18–24	57,931 (7.9)	26,218 (5.8)
25–39	338,539 (46.1)	196,260 (43.3)
40–49	133,140 (18.1)	86,834 (19.2)
50–64	160,038 (21.8)	111,727 (24.7)
≥65	43,557 (5.9)	31,393 (6.9)
Unknown	11 (—)	0 (—)
**Race and ethnicity^¶^**		
AI/AN, non-Hispanic	2,762 (0.4)	1,602 (0.4)
Asian, non-Hispanic	49,675 (7.4)	30,624 (7.3)
Black or African American, non-Hispanic	83,014 (12.4)	48,472 (11.5)
NH/OPI, non-Hispanic	1,730 (0.3)	1,004 (0.2)
White, non-Hispanic	341,419 (51.2)	228,158 (54.2)
Hispanic or Latino	151,647 (22.7)	88,554 (21.0)
Multiple/Other, non-Hispanic	37,063 (5.6)	22,903 (5.4)
Unknown	67,200 (—)	31,527 (—)
U.S. Census Bureau region**		
Northeast	180,986 (24.8)	100,575 (22.3)
Midwest	90,297 (12.4)	56,163 (12.5)
South	197,700 (27.0)	133,002 (29.5)
West	261,936 (35.8)	160,457 (35.6)
**Urbanicity^††^**		
Urban	563,246 (82.3)	346,002 (81.0)
Suburban	108,810 (15.9)	73,126 (17.1)
Rural	12,646 (1.8)	7,919 (1.9)
Unknown	49,808 (—)	25,797 (—)
**Location of vaccine administration**		
Public health provider (public health clinic)	246,938 (39.5)	139,766 (36.2)
Commercial vaccination service provider	84,029 (13.4)	64,334 (16.6)
Medical practice	63,682 (10.2)	41,627 (10.8)
Hospital	55,332 (8.8)	25,192 (6.5)
Public health provider (FQHC)	38,780 (6.2)	26,047 (6.7)
Health center (community)	22,641 (3.6)	15,064 (3.9)
Health center (other)	20,464 (3.3)	12,494 (3.2)
Pharmacy	19,859 (3.2)	14,085 (3.6)
Other	73,845 (11.8)	47,856 (12.4)
Unknown	108,940 (—)	66,379 (—)
**Administration route^§§^**		
Intradermal	288,979 (45.9)	346,410 (85.7)
Subcutaneous	318,805 (50.6)	47,359 (11.7)
Other	21,791 (3.5)	10,304 (2.6)
Unknown	75,794 (—)	30,250 (—)
**Estimated coverage of persons at risk, %^¶¶^**	36.7	22.7
**Range of jurisdiction values**	7–95	5–66

**Sources**: CDC Immunization Data Lake and AtlasPlus.

**Abbreviations**: AI/AN = American Indian or Alaska Native; FQHC = Federally Qualified Health Center; mpox = monkeypox; MSM = men who have sex with men; NCHS = National Center for Health Statistics; NH/OPI = Native Hawaiian or other Pacific Islander; PrEP = HIV preexposure prophylaxis.

* Data current as of March 7, 2023.

^†^ Percentages calculated using nonmissing data.

^§^ 2,257 doses were reported as third, fourth, fifth, sixth, or seventh doses.

^¶^ Combined information about a person’s race and Hispanic ethnicity. Persons reporting Hispanic ethnicity were categorized as Hispanic or Latino (Hispanic) and might be of any race; persons reporting non-Hispanic ethnicity were categorized as White, Black, Asian, AI/AN, NH/OPI, or other/multiracial (more than one race category selected) based on race; persons with missing data for either race or ethnicity were categorized as unknown race and ethnicity (9.1% of first dose recipients and 7.0% of second dose recipients did not have race and ethnicity data reported).

** https://www2.census.gov/geo/pdfs/maps-data/maps/reference/us_regdiv.pdf. As of January 31, 2023, vaccine allocations (vials) for each U.S. Census Bureau region were as follows: 275,906 (Northeast), 169,419 (Midwest), 428,686 (South), and 302,895 (West) (https://aspr.hhs.gov/SNS/Pages/JYNNEOS-Distribution.aspx). A total of 6,246 vaccine recipients were in U.S. territories and freely associated states and were not categorized in a U.S. Census Bureau region.

^††^ Urbanicity was classified based on the vaccine recipient’s county of residence using the NCHS Urban-Rural Classification Scheme for Counties 2013: urban includes large central metropolitan, medium metropolitan, and small metropolitan counties; suburban includes large fringe metropolitan counties; rural includes micropolitan and noncore counties. A total of 69,441 vaccine recipients had an unknown or missing county of residence; 6,264 vaccine recipients were in U.S. territories and freely associated states and were not categorized as urban, suburban, or rural.

^§§^ Does not include vaccine administrations for jurisdictions reporting aggregate data. Analyses do not include vaccine doses reported by Texas and vaccine doses administered to recipients aged <18 years reported by Idaho because of aggregate reporting to CDC.

^¶¶^ Estimated mpox vaccination coverage is the proportion of all persons aged ≥13 years who have been vaccinated divided by the population recommended to receive the vaccine. The denominator, representing the population at increased risk for *Monkeypox virus* exposure, is estimated as the number of MSM who are eligible for PrEP (among those aged ≥16 years) plus the number of MSM living with HIV (among those aged ≥13 years) in each jurisdiction. These data are publicly available via CDC AtlasPlus (https://gis.cdc.gov/grasp/nchhstpatlas/tables.html) as of 2021 (PrEP-eligible MSM) and 2020 (MSM with HIV). This estimated population has been increased by 25% for each jurisdiction to account for additional persons eligible for vaccination (e.g., cis-female or transgender partners of MSM and close contacts of persons with known or suspected mpox). Data current as of March 16, 2023.

The distribution of administration routes differed between the first and second doses. Overall, 45.9% of first doses were administered intradermally, 50.6% were administered subcutaneously, and 3.5% were administered by other routes. Among the second doses, 85.7% were administered intradermally, 11.7% subcutaneously, and 2.6% by other routes. The change from predominantly subcutaneous to predominantly intradermal administration occurred after the week of August 20, 2022 (Figure 1).

**FIGURE 1:**
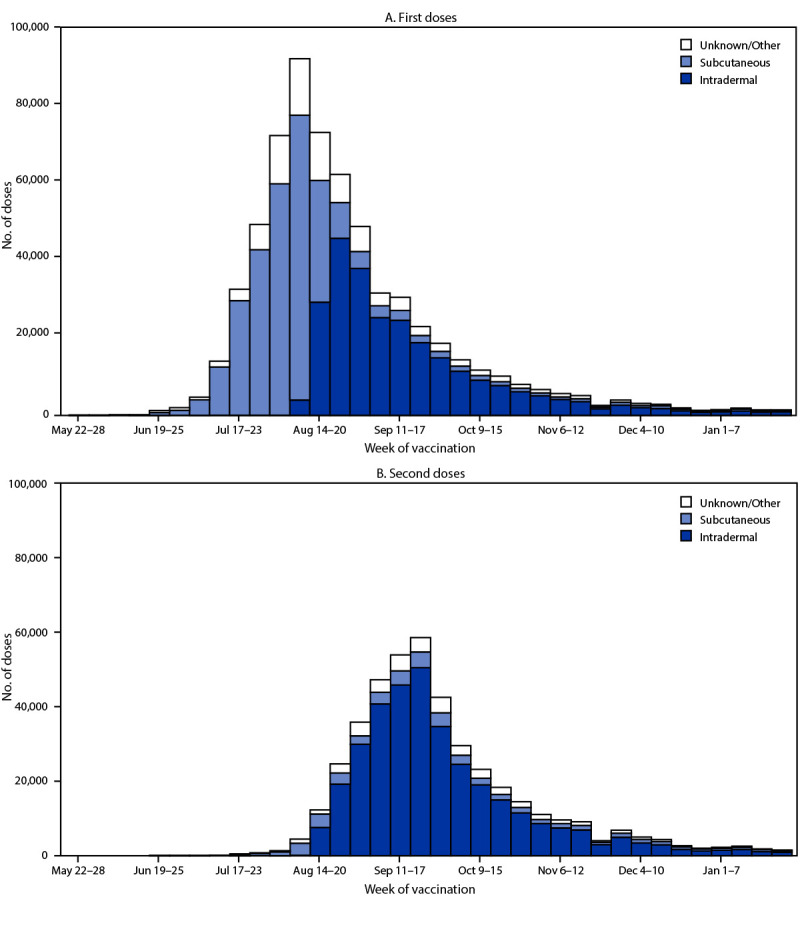
Route of administration of first and second JYNNEOS vaccine doses, by week of vaccination* — United States, May 22, 2022–January 28, 2023 **Source:** CDC Immunization Data Lake. * Data reported to CDC as of 4:00 a.m. Eastern Daylight Time on March 7, 2023. Weeks in which n<30 are not shown. Data does not include vaccine administration for jurisdictions reporting aggregate data. Analyses do not include vaccine administration reported by Texas or reported for recipients aged <18 years by Idaho because of aggregate reporting to CDC.

National first and second dose JYNNEOS coverage among persons at increased risk for mpox were estimated to be 36.7% and 22.7%, respectively. Coverage estimates varied by jurisdiction, ranging from 7.4% (West Virginia) to 94.8% (DC) for first dose coverage and 4.6% (West Virginia) to 66.2% (DC) for second dose coverage. Jurisdictions estimated to have achieved ≥50% coverage for first dose (partial vaccination) were DC (94.8%), New York City (88.8%), California (61.4%), Rhode Island (58.9%), Massachusetts (53.9%), and New York (excluding New York City) (50.1%) (Figure 2).

**FIGURE 2:**
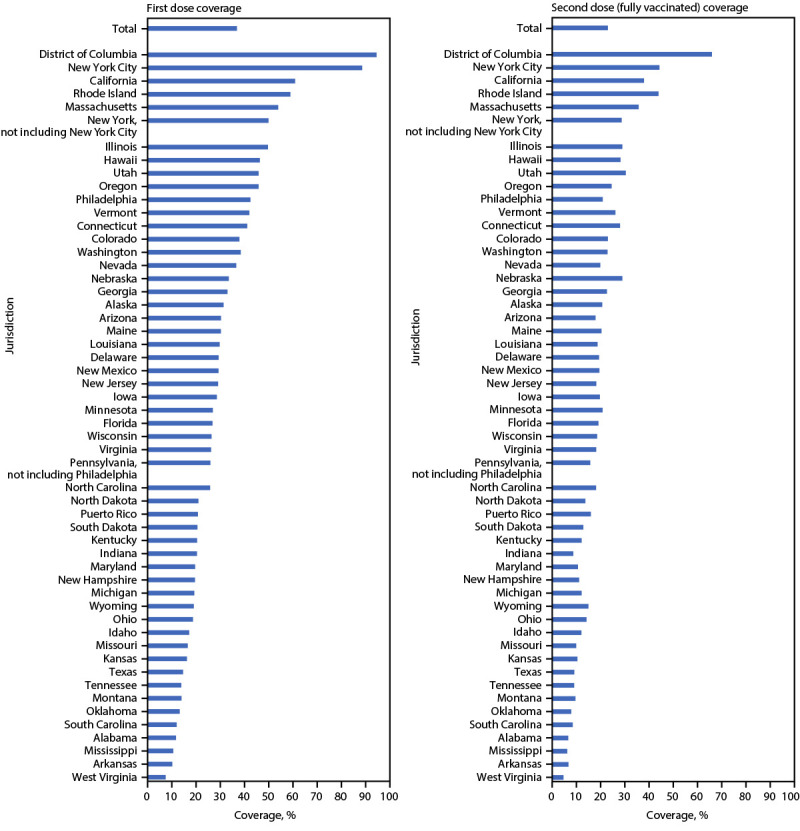
First and second* JYNNEOS vaccination coverage estimates, by jurisdiction^†^ — United States, May 22, 2022–January 31, 2023^§^ **Sources**: CDC Immunization Data Lake and AtlasPlus. * Fully vaccinated is defined as receipt of 2 JYNNEOS doses on different days, irrespective of time interval, with the second dose received ≥14 days earlier. ^†^ Residency was ascertained by vaccine recipient self-report; in the absence of a residential address, the location of vaccination was used in some locations. New York City and Philadelphia were allocated and report vaccine doses to CDC separately from the rest of New York and Pennsylvania and are therefore included as separate jurisdictions in this report. ^§^ Data reported to CDC as of March 16, 2023. U.S. Virgin Islands, Guam, and the Northern Mariana Islands are not included in coverage estimates because of data suppression restrictions.

## Discussion

The national public health response strategy for mpox has resulted in administration of >1 million JYNNEOS vaccine doses. Although approximately one in eight first dose recipients were reported to be Black, this group accounts for approximately one in three mpox cases^††††^ (*9*), underscoring the importance of deploying strategies, including vaccination, that advance health equity among populations most affected by mpox. The smaller dose needed for intradermal administration increased the available vaccine supply, with each vial providing up to 5 doses compared with a single subcutaneous dose (*7*). The intradermal administration authorization by FDA was widely implemented within 2 weeks of issuance of the EUA in early August 2022.

Estimating vaccination coverage among persons at risk is useful for implementing and assessing public health action. Mpox vaccination coverage varied widely by jurisdiction. Three of the six jurisdictions estimated to have 1-dose coverage rates of ≥50% were also among the jurisdictions with the highest case counts (New York City, California, and New York [excluding New York City]).^§§§§^ Twenty-two jurisdictions are estimated to have ≤25% first-dose coverage. DC is the only jurisdiction estimated to have achieved >50% 2-dose coverage. Reasons for low or high coverage were not assessed in this analysis, but potential reasons for low coverage in some jurisdictions could include lower vaccine accessibility and awareness, fewer vaccine providers, lower vaccine confidence and demand, and concern about stigma. CDC recommends that all persons eligible for mpox vaccination get vaccinated; however, national vaccination coverage targets have not been established. Vaccine allocation and distribution were prioritized by risk for exposure to *Monkeypox virus* and for areas with higher case counts, making more doses available in highly affected jurisdictions during the period of peak vaccine demand.^¶¶¶¶^

The findings in this report are subject to at least four limitations. First, coverage estimates include assumptions based on national data applied to subnational jurisdictions. For example, the increase by 25% of the vaccine-eligible population might not be appropriate for each jurisdiction. Consequently, coverage estimates for some jurisdictions might be biased. Second, residence information was self-reported and might be incomplete; in cases for which residence was not provided to the vaccination site, the site’s location might have been used. Overall, among vaccinations reported to CDC, residence data were missing for 8%. Third, some vaccination-related data elements are not available for all jurisdictions; therefore, national estimates for some characteristics might not include all jurisdictions. For example, some jurisdictions do not report recipients’ sex or race and ethnicity. Finally, information on sexual orientation and gender identity are not routinely collected during vaccine administration, and existing IIS systems do not include those variables; therefore, it is not possible to assess how vaccine recipients identify.

Although the number of mpox cases has decreased sharply, much is unknown about the risk for potential reintroduction of the virus and resurgence of disease in the United States. Thus, the need to improve vaccination coverage among populations at risk, increase vaccine equity, and increase the number of persons fully vaccinated (*10*) remain important public health goals to prevent or minimize the impact of a resurgence of mpox. Given the increased protection provided by full vaccination compared with partial vaccination (*3*), providing second doses to those who are partially vaccinated should also be prioritized. Continued targeted local outreach to persons at increased risk for mpox and to disproportionately affected groups and community partners to address inequities is recommended. Ongoing access to vaccine administration data from jurisdictions is important for public health decision-making and improving equitable vaccination coverage among those at risk.

**Summary**

**What is already known about this topic?**

JYNNEOS vaccine is effective in preventing monkeypox (mpox) among persons at risk.

**What is added by this report?**

During the current multinational outbreak, U.S. 1- and 2-dose vaccination coverage reached an estimated 37% and 23%, respectively, among persons at risk, with wide variation among jurisdictions. The predominant administration route switched from subcutaneous to intradermal after the week of August 20, 2022, in accordance with Food and Drug Administration recommendations.

**What are the implications for public health practice?**

Despite administration of >1 million vaccine doses, only 23% of the at-risk population has been fully vaccinated. Targeted outreach and continued access to and availability of mpox vaccines to persons at risk is important to help prevent and minimize the impact of a resurgence of mpox.

## Footnotes

*
https://www.cdc.gov/poxvirus/mpox/response/2022/world-map.html

† Residency was ascertained by vaccine recipient self-report; in the absence of a residential address, the location of vaccination was used in some locations. New York City and Philadelphia were allocated and report vaccine doses to CDC separately from the rest of New York and Pennsylvania and are therefore included as separate jurisdictions in this report.

§ 2,257 doses were reported as third, fourth, fifth, sixth, or seventh doses.

¶
https://www.cdc.gov/poxvirus/mpox/response/2022/index.html

**
https://www.cdc.gov/poxvirus/mpox/vaccines/index.html

†† Assessing vaccination based on gender identity was not possible because this information is not routinely collected during vaccine administration, and existing IIS systems do not include this variable.

§§ U.S. Virgin Islands, Guam, and the Northern Mariana Islands are not included in coverage estimates because of data suppression restrictions.

¶¶ Fully vaccinated is defined as receipt of 2 JYNNEOS doses on different days, irrespective of time interval, with the second dose received ≥14 days earlier.

***
https://gis.cdc.gov/grasp/nchhstpatlas/tables.html

†††
https://www.cdc.gov/vaccines/hcp/acip-recs/vacc-specific/smallpox.html

§§§ Estimated population calculation based on consensus within CDC mpox response and is consistent with the mpox vaccine allocation strategy. https://aspr.hhs.gov/Mpox/Pages/default.aspx

¶¶¶ 45 C.F.R. part 46, 21 C.F.R. part 56; 42 U.S.C. Sect. 241(d); 5 U.S.C. Sect. 552a; 44 U.S.C. Sect. 3501 et seq.

**** Broad category used by IIS, includes public and private service providers.

††††
https://www.cdc.gov/poxvirus/mpox/response/2022/demographics.html

§§§§
https://www.cdc.gov/poxvirus/mpox/response/2022/us-map.html

¶¶¶¶
https://aspr.hhs.gov/SNS/Pages/JYNNEOS-Distribution.aspx

## References

[1] CDC. Mpox: vaccination. Atlanta, GA: US Department of Health and Human Services, CDC; 2023. Accessed March 6, 2023. https://www.cdc.gov/poxvirus/mpox/interim-considerations/overview.html

[2] CDC. Mpox: JYNNEOS vaccine. Atlanta, GA: US Department of Health and Human Services, CDC; 2023. Accessed February 2, 2023. https://www.cdc.gov/poxvirus/mpox/interim-considerations/jynneos-vaccine.html

[3] Payne AB, Ray LC, Cole MM, Reduced risk for mpox after receipt of 1 or 2 doses of JYNNEOS vaccine compared with risk among unvaccinated persons—43 U.S. jurisdictions, July 31–October 1, 2022. MMWR Morb Mortal Wkly Rep 2022;71:1560–4. 10.15585/mmwr.mm7149a5 36480479 PMC9762900

[4] Payne AB, Ray LC, Kugeler KJ, Incidence of monkeypox among unvaccinated persons compared with persons receiving ≥1 JYNNEOS vaccine dose—32 U.S. jurisdictions, July 31–September 3, 2022. MMWR Morb Mortal Wkly Rep 2022;71:1278–82. 10.15585/mmwr.mm7140e3 36201401 PMC9541026

[5] Wolff Sagy Y, Zucker R, Hammerman A, Real-world effectiveness of a single dose of mpox vaccine in males. Nat Med 2023;29:748–52. 10.1038/s41591-023-02229-3 36720271 PMC9930701

[6] Food and Drug Administration. Fact sheet for healthcare providers administering vaccine: emergency use authorization of JYNNEOS (smallpox and monkeypox vaccine, live, non-replicating) for prevention of monkeypox disease in individuals determined to be at high risk for monkeypox infection. Silver Spring, MD: US Department of Health and Human Services, Food and Drug Administration; 2022. Accessed March 16, 2023. https://www.fda.gov/media/160774/download.

[7] Brooks JT, Marks P, Goldstein RH, Walensky RP. Intradermal vaccination for monkeypox—benefits for individual and public health. N Engl J Med 2022;387:1151–3. 10.1056/NEJMp2211311 36044621

[8] CDC. Mpox: science brief: detection and transmission of mpox (formerly Monkeypox) virus during the 2022 Clade IIb outbreak. Atlanta, GA: US Department of Health and Human Services, CDC; 2023. Accessed February 21, 2023. https://www.cdc.gov/poxvirus/mpox/about/science-behind-transmission.html

[9] Kava CM, Rohraff DM, Wallace B, Epidemiologic features of the monkeypox outbreak and the public health response—United States, May 17–October 6, 2022. MMWR Morb Mortal Wkly Rep 2022;71:1449–56. 10.15585/mmwr.mm7145a4 36355615 PMC9707350

[10] Kriss JL, Boersma PM, Martin E, Receipt of first and second doses of JYNNEOS vaccine for prevention of monkeypox—United States, May 22–October 10, 2022. MMWR Morb Mortal Wkly Rep 2022;71:1374–8. 10.15585/mmwr.mm7143e2 36301741 PMC9620573

